# Virulence of Pertactin-Negative *Bordetella pertussis* Isolates from Infants, France

**DOI:** 10.3201/1903.121475

**Published:** 2013-03

**Authors:** Hélène Bodilis, Nicole Guiso

**Affiliations:** Institut Pasteur, Paris, France;; Centre National de la Recherche Scientifique (CNRS)

**Keywords:** *Bordetella pertussis*, acellular vaccines, bacteria, coccobacillus, immunization, vaccination, vaccine, pertactin

## Abstract

*Bordetella pertussis* isolates that do not express pertactin (PRN) are increasing in regions where acellular pertussis vaccines have been used for >7 years. We analyzed data from France and compared clinical symptoms among infants <6 months old infected by PRN-positive or PRN-negative isolates. No major clinical differences were found between the 2 groups.

*Bordetella pertussis* and *B. parapertussis* are closely related bacterial species, and both cause whooping cough. As early as 1959, whole-cell pertussis vaccine was used intensively in France for primary vaccination of infants at 3–5 months of age and for the first booster at 24 months (*1*). This vaccine program resulted in a dramatic decrease in the incidence of pertussis among young children. Acellular pertussis vaccines (2- and 3–component vaccines) were introduced in 1998 as boosters for vaccinated adolescents and were rapidly adopted for primary vaccination of infants. These vaccines replaced whole-cell pertussis vaccines in 2005, changing herd immunity by specifically targeting the virulence of the bacteria (*2**,**3*). 

Since 1996, in France, an active hospital-based surveillance network has performed whooping cough surveillance. The network comprises 42 pediatric hospitals, which participate on a voluntary basis; the National Reference Centre, which is located in the laboratory of the Molecular Prevention and Therapy of Human Diseases Unit at Institut Pasteur; and the French Institute for Public Health surveillance (*3*–*4**)*. Participating pediatricians complete a standardized form for every child suspected to have whooping cough. Microbiologists list culture and PCR results and send the clinical isolates to the National Reference Centre for validation of the results. This system of data collection has been unchanged since establishment of the network; data collected is used to analyze trends over time (*3**–**4**)*. 

We have analyzed the evolution of the bacterial population under vaccine pressure, using pulsed-field gel electrophoresis, genotyping, microarrays, and tests for virulence factor expression (*5*–*9*). Immunity induced by the whole-cell pertussis vaccine controlled the circulation of vaccine-type isolates but not all types of isolates (*5*,^6^). The isolates remaining in circulation are as virulent as those circulating during the prevaccine era (*7*–*9*). Since the introduction of acellular pertussis vaccines, the number of *B. pertussis* and *B. parapertussis* isolates collected that do not express pertactin (PRN), which is used as a vaccine antigen (*7**–**11*), has steadily increased. The proportion of PRN-negative (PRN–) isolates to the total number of isolates collected each year increased from 2% in 2005 to 14% in 2012 (*8*), indicating that PRN– isolates are transmissible. Studies using animal and cellular models of infection indicate that these PRN– isolates are as virulent as those expressing PRN (PRN+) (*7**–**9**).* However, an analysis and comparison of the clinical symptoms induced by infection with PRN– and PRN+ isolates in infants convey direct information on this strictly human disease. Here, we report a preliminary retrospective comparison of the clinical symptoms of infants <6 months old in France who were infected by PRN– isolates and clinical symptoms of those infected with PRN+ isolates during 2004–2011. 

## The Study

For the purpose of this study, we used a questionnaire that was more detailed than the one in the standardized form from the hospital-based surveillance program. The questionnaire, including the list of variables described in Table 1, was sent to pediatricians who voluntarily participated. We compared surveys for each patient infected by a PRN– isolate with 2 or 3 randomly selected standardized forms that had been completed by pediatricians and that described patients <6 months of age who were infected by PRN+ isolates during the same period. We sent 68 questionnaires (20 for PRN– isolates, 48 for PRN+ isolates). We received 60 completed questionnaires (40 for infants infected with a PRN+ isolate, 20 for infants with a PRN– isolate).

**Table 1:** Characteristics and clinical signs and symptoms of patients <6 mo of age infected with *Bordetella pertussis* isolates negative or positive for pertactin, France, 2004–2011*

Variable	Pertactin-negative isolate, n = 20	Pertactin-positive isolate, n = 40	p value
Male sex, %	60	50	0.46†
Age, d (range)	66 (16–147)	61 (23–132)	NA
Year of illness, %			NA
2004	5	2.5	NA
2005	10	7.5	NA
2006	5	5	NA
2007	15	15	NA
2008	5	7.5	NA
2009	25	35	NA
2010	5	22.5	NA
2011	30	5	NA
Previous vaccination	4/19 (21.05)	8/39 (20.51)	0.96†
Vaccinated according to recommendations	n = 19	n = 39	NA
Yes	2 (10.53)	4 (10.26)	1.0‡
No	5 (26.32)	10 (26.54)	NA
Not eligible (<2 mo of age)	12 (63.16	25 (64.10)	NA
Time from onset of signs and symptoms to sample collection, d (range)	14.6 (1–37), n = 13	9.9 (2–35), n = 33	0.04§
Signs and symptoms			
Nocturnal cough	6/7 (86)	19/22 (86)	1‡
Paroxysmal cough	16/16 (100)	35/36 (97)	1‡
Syncope	4/9 (44)	8/25 (32)	0.69‡
Vomiting	3/11 (27)	15/29 (52)	0.29‡
Loss of weight	5/10 (50)	10/25 (40)	0.71‡
Whoop	5/8 (62)	6/14 (43)	0.66‡
Apnea	5/8 (62)	11/24 (46)	0.68‡
Fever	2/11 (18)	3/31 (10)	0.59‡
Bradycardia	4/7 (57)	14/20 (70)	0.6‡
Atypical cough	1/9 (11)	6/17 (35)	0.36‡
Cyanosis/desaturation	9/11 (82)	20/25 (80)	1‡
Deterioration of general condition	4/9 (44)	9/28 (32)	0.77‡
Malignant pertussis	0/9	1/28 (4)	NA
Hyperlymphocytosis	8/9 (89)	17/23 (74)	0.64‡
Hospitalization	16/18 (89)	36/39 (92)	0.65‡
Duration of hospitalization, d (range)	12.6 (1–45), n = 9	16.6 (1–60), n = 28	0.18§
Intensive care	5/10 (50)	13/30 (43)	0.73‡
Duration of intensive care, d (range)	8.2 (2–21), n = 5	5.4 (1–14), n = 12	0.24§

*Values are (no. patients with variable/no. of patients with data) except as indicated. NA, not applicable. †χ^2^ test. ‡Fisher exact test. §Mann-Whitney U test.

The available anonymous variables analyzed are shown in Table 1. To compare percentages, we used the χ^2^ or Fisher exact test if n<5. To compare means, we used the Mann-Whitney U test. There were no substantial differences in distribution of PRN– and PRN+ isolates among patients in the 2 groups in terms of sex and age (60% of infants infected with PRN– isolates were boys, as were 50% of those infected with PRN+ isolates; the mean ages of infants in each group were 66 and 61 days, respectively). There was an even distribution of PRN– and PRN+ isolates among the infants across the years studied. Forty-six infants had received no pertussis acellular vaccine, and 11 had received 1 dose. One child >4 months of age received a second dose 4 days before the onset of symptoms. According to information compiled from the survey that used the standardized form, 21.05% of PRN– patients and 20.51% of PRN+ patients had been vaccinated. None of the children had received 3 doses. In each group, ≈10% of infants received vaccinations as scheduled for their age (1 dose of vaccine for each infant). The duration of hospitalization or stay in intensive care was shorter for the group of infants infected with a PRN– isolate, but the difference was not significant (p = 0.18 vs. p = 0.24). The differences found between the 2 groups of infants in terms of the classical symptoms (apnea, vomiting, paroxysmal cough, whoop, bradycardia, and hyperlymphocytosis) were not significant (p = 0.68, p = 0.29, p = 1, p = 0.66, p = 0.6, and p = 0.64, respectively). The only significant difference (p = 0.04) was that the time between the beginning of the cough and hospitalization was longer for infants infected with a PRN– isolate; this finding might reflect less severe disease in this group.

We calculated delay of transmission as the time of onset of coughing by the first member of a household to that by the case-patient. The median delay of transmission was 14.5 and 14.0 days, respectively, in PRN– and PRN+ groups. Among the documented cases, *B. pertussis* was transmitted to the infant by a household member in 84% of the PRN– cases and 91% of the PRN+ cases.

Vaccination was associated with less severe clinical symptoms (Table 2): the proportion of hospitalizations in intensive care units was significantly lower in the vaccinated group (p = 0.001). Clinical symptoms, such as apnea, syncope, cyanosis, and deterioration of general condition, were also less frequent in the vaccinated group (Table 2). This confirms previous findings (*12*) indicating that infants who receive 1 or 2 doses of pertussis vaccine are protected to some extent.

**Table 2:** Clinical signs and symptoms of vaccinated and unvaccinated pertussis patients, France, 2004–2007

Characteristics	No. patients/no. with characteristic (%)		p value*
	Not vaccinated, n = 46	Received 1–2 doses, n = 12	
Intensive care admission	18/34 (53)	0/11 (0)	0.001
Apnea	15/25 (60)	1/7 (14)	0.08
Cyanosis/desaturation	24/28 (86)	5/8 (62)	0.167
Syncopal episodes	12/26 (46)	0/8 (0)	0.03
Bradycardia	15/21 (71)	3/6 (50)	0.367
Deterioration of general condition	12/29 (41)	2/8 (25)	0.68
Malignant pertussis	1/29 (3)	0/8 (0)	1.0


*By Fisher exact test.

## Conclusions

These preliminary data are consistent with those we obtained using murine and cellular models (*8**,**9*). Although the number of infants included in this study is small, we could detect no major difference between the 2 groups; this finding suggests that PRN– isolates are as virulent as PRN+ isolates. This conclusion is also in agreement with data obtained during a clinical trial performed in Italy (*13*). We recommend the continuation of such analyses, and close collaboration of clinicians and microbiologists, to follow the evolution of *B. pertussis* subspecies in terms of virulence. This will help identify strategies to overcome increased adaptive herd immunity induced by acellular pertussis vaccines.
